# Editorial—Health Is a Duty

**Published:** 1893

**Authors:** 


### EDITOR'S OUTLOOK.

### HEALTH IS A DUTY.

When we announced, as a starting point in the first number of our Journal, in January, 1854, that a man ought to be well, and that being sick was an implied wrong, no doubt it appeared to many rather a rigid doctrine, to wit, that it was a sin to be sick.

But men of reflection will not be long in coming to the conclusion, that if it is not so in some cases, it is so in a vast multitude of instances; and a practical man may benefit himself largely, if he be also conscientious, by inquiring, when incapacitated from discharging the duties of life by illness. "Is it my fault?" A servant who has cut off his hand to avoid labor, has certainly done deliberate wrong to the person to whom he justly owed labor.

And although we may deliberately make ourselves sick, yet, if it is done through gross inattention or ignorance, the degree of criminality is but a short distance from the other.

The "Duty of Health" was considered of sufficient importance to make it the subject of a sermon by Dr. Lee, of the University of Edinburgh, before the British Queen, on the 11th of October, 1857, which sermon was thought worthy of being printed, and was so ordered by her Majesty.

The ordinances of Moses embodied a very complete code of health, and the religion of the New Testament certainly tends to the same great end.

Dr. Lee says: "It is estimated that one hundred thousand people die annually in England of preventable diseases." In the same proportion, more than a million and a quarter must die annually from the same causes in Europe. Probably not fewer than four hundred thousand men were killed during the Russian war. But, during the same period ten times as many died in Europe alone, from preventable diseases. The slaughter of four million persons in three years, in a war against the laws of health. So appalling a fact is surely deserving the earnest attention, not only of governors, politicians, and philanthropists, but of all men who profess Christianity, and especially those who are appointed to teach it; because the laws of health, through disobedience to which such multitudes perish, are God's laws, for he not only ordained them, but he executed them, impartially and universally before our eyes, and upon ourselves.

We desire to especially call attention to our offer found in our advertising department. We have found by experience that such a premium offer as the 30 volumes of Chambers Encyclopedia was largely appreciated and taken advantage of. Our piano offer, we feel, will be likewise appreciated and that earnest work will be indulged in by those who desire an $800 piano *free*. Remember the contest closes April 1st, 1894.

Our winter Recreation Bureau will be inaugurated in our December number. Meantime, we invite all hotel proprietors at the various winter resorts, to send in circulars of their hotels *at once*, for use in our Bureau.

